# Effectiveness of a village-based intervention for depression in community-dwelling older adults: a randomised feasibility study

**DOI:** 10.1186/s12877-020-1495-2

**Published:** 2020-03-04

**Authors:** In Mok Oh, Maeng Je Cho, Bong-Jin Hahm, Byung-Soo Kim, Jee Hoon Sohn, Hye Won Suk, Bu Young Jung, Hye Jung Kim, Hyeon A. Kim, Ki Bok Choi, Da Hye You, Ah. Reum Lim, In Ok Park, Jeung Hyuck Ahn, Hee Lee, Yeon Hee Kim, Mi Ra Kim, Jee Eun Park

**Affiliations:** 1 Department of Psychiatry and Behavioral Science, Seoul National University College of Medicine and Seoul National University Hospital, 101, Daehak-ro, Jongno-gu, Seoul, 110-744 South Korea; 2 Department of Psychiatry, School of Medicine, Kyungpook National University, 80, Daehak-ro, Buk-gu, Daegu, Republic of Korea; 3 Department of Psychology, Sogang University, 35, Baekbeom-ro, Mapo-gu, Seoul, Republic of Korea; 4 Yeoncheon Commnity Mental Health Center, 95, Eundaeseong-ro, Jeongok-eup, Yeoncheon-gun, Gyeonggi-do, Republic of Korea

**Keywords:** Old-aged, Community-based, Intervention, Late-life depression, Suicide

## Abstract

**Background:**

Although a focus on late-life depression may help preventing suicide in older adults, many older people, especially those living in rural areas, have relatively low accessibility to treatment. This study examined the feasibility and effectiveness of a village-based intervention for depression targeting older adults living in rural areas.

**Methods:**

A community-based randomised pilot trial was performed in two small rural villages in South Korea. Two villages were randomly selected and assigned to the intervention or active control group; all older adults living in the two villages (*n* = 451) were included in the intervention program or received standard Community Mental Health Service (CMHS) care, and the effectiveness of the program was examined using representative samples from both groups (*n* = 160). The 12-week intervention included case management according to individual risk level and group-based activities. Healthy residents living in the intervention village who played major roles in monitoring at-risk older individuals were supervised by CMHS staff. The score on the Korean version of the Geriatric Depression Scale-Short Form (SGDS-K) was the primary outcome, while social network, functional status, and global cognitive function were secondary outcomes. Linear mixed models including the factors of intervention group, time, and their interaction were used to examine group differences in changes in primary and secondary outcomes from baseline to follow up.

**Results:**

Overall, there was no significant group × time interaction with respect to the SGDS-K score, but older individuals with more depressive symptoms at baseline (SGDS-K ≥ 6) tended to have a lower likelihood of progressing to severe depression at post-intervention. The social network was strengthened in the intervention group, and there was a significant group × time interaction (*F*[*df1, df2*], 5.29 [1, 153], *p* = 0.023).

**Conclusion:**

This study examined a 12-week village-based intervention for late-life depression in which the CMHS helped village-dwellers deal with late-life depression in their communities. Although the intervention improved social interactions among older adults, it did not reduce depressive symptoms. Further studies including more rural villages and long-term follow up are needed to confirm the effectiveness of this prevention program.

**Trial registration:**

NCT04013165 (date: 9 July 2019, retrospectively registered).

## Background

The suicide rates in older adults in South Korea are much higher than those in other Asian and Western countries; the rate was 47.7 per 100,000 older Koreans in 2017 [1], compared with 13.8 in the United States, 22.9 in the European Union, and 21.1 in Japan [2, 3]. It is also roughly double that of the younger population in Korea (24.3 per 100,000) [1], and is now regarded as a major social issue. In Korea, suicide rates are higher in rural areas than in urban regions [4], which might be due to the large numbers of elderly people living in rural areas. Suicide prevention strategies tailored by age and residential area are needed; however, only a few trials have examined this issue in older adults living in rural areas [5].

Among the risk factors for suicide in older people, depression is an important modifiable factor [6–8], and has been a target of several suicide prevention trials [9–11]. However, late-life depression is usually under-recognized and under-treated compared with depression in younger adults, because of the propensity for masked depression and comorbid physical illnesses [12–14]. Therefore, previous interventions have focused on the surveillance of depression by primary care physicians, or have targeted older adults who already had a clinical diagnosis of depression [9–11]. However, small rural areas usually have insufficient medical services, including a lack of general physicians (GPs) and psychiatrists. Furthermore, a meta-analysis showed that GPs were less successful in identifying depression in older adults than in younger people [14], whereas a recent review reported that community outreach services effectively reduced the risk of suicide [15].

Therefore, this study tested the feasibility of a self-sustainable community-based intervention for depression, targeting older adults living in rural villages. We developed a 12-week program that was implemented by the community mental health service (CMHS) team; in this program, community-dwelling healthy adults played the main role in preventing depression in at-risk older adults. Rural areas have some beneficial features, such as relatively small physical and psychological distances among individuals; this may facilitate socializing among neighbors. Although 243 CMHS centers have been installed throughout Korea, each service team has multiple roles, caring not only for depressed older people but also for individuals with other chronic mental disorders. Therefore, we developed a short-term, sustainable depression intervention for small rural areas that encouraged the participation of healthy residents who could continue in their depression prevention role after the program ended.

This pilot study investigated the efficacy of a community-based multilevel intervention for late-life depression in two small rural areas in Korea. Two villages were allocated randomly to the intervention group and active control; all older adults living in the two villages were included in either the intervention program or received standard CMHS care, and the effectiveness of the protocol was assessed in representative older adults according to age- and sex-stratified random sampling.

## Methods

### Study design

A community-based randomised pilot trial was conducted to evaluate the feasibility and the effectiveness of a 12-week village-based intervention for late-life depression. Yeoncheon County is a rural area with a population of 44,187 (10,735 older adults), and has two towns and 96 small villages [16]. Two villages in Yeoncheon were randomly selected as the intervention group (Gungpyeong) and active control (Baekgui), respectively. These two villages contain similar numbers of older adults [16], and the geographical distance between them is 30-min travel by car. The trial was mainly coordinated by the Yeoncheon CMHS team from June 2017 to March 2018. Our late-life depression intervention was developed for pragmatic use in a community setting, and the CMHS staff, including nurses and social workers, administered the protocol as study assistants. Although this study was not blinded, the residents in the two villages were not informed about the group allocations. Furthermore, although most of the assessments were performed by researchers who were also blinded to the group allocation, some were done by the staff who managed the program.

### Participants

Although all older residents in the two villages (*n* = 451) received either the intervention treatment or standard care, the effectiveness of the protocol was examined in a subset of representative older residents selected using an age- and sex-stratified random sampling method based on population data for Yeoncheon for 2015 (*n* = 160) [16]. At screening, subjects with significant sensory deficits or medical illnesses that would substantially affect delivery of assessments were excluded. The sample size required for measurement of the primary outcome measure, i.e., scores on the Korean version of the Geriatric Depression Scale – Short Form (SGDS-K) [17], was determined a priori based on a previous preventive study; a sample size of 142 (71 per group) was needed to detect an inter-group difference in change in SGDS-K score with a power of 0.80 and a two-sided alpha of 0.05 (*Cohen’s d* = 0.48) [9]. Based on our previous experience, a 10% attrition rate was projected, which increased the required sample size to 160 (80 per group).

All participants were fully informed of the study aims and methods and informed consent was obtained before the screening interview. The study was approved by the Institutional Review Board of Seoul National University Hospital (IRB No. 1807–135-961) and was registered in a clinical trial registry (registration NCT04013165).

### Intervention

**Fig. 1:**
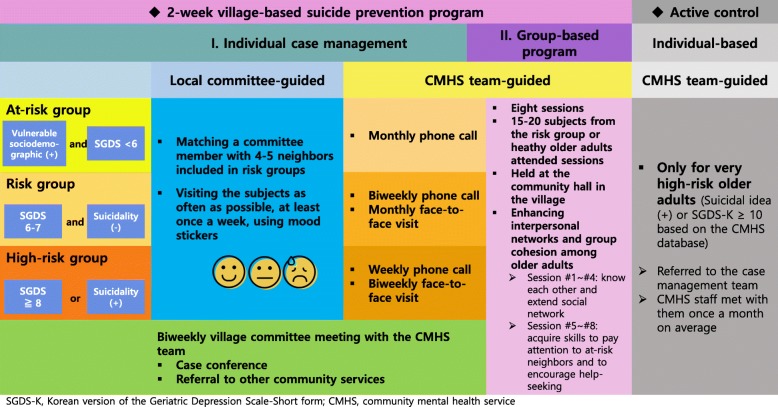
The 12-week protocol for the village-based intervention for late-life depression. SGDS-K, Korean version of the Geriatric Depression Scale-Short form; CMHS, community mental health service

A 12-week village-based intervention for late-life depression was tested, with the aim of reducing depressive symptoms and suicidal risk via a multilevel strategy delivered within the existing community-based mental health care framework. The program was conducted by CMHS staff and healthy residents living in the village; 12 healthy residents voluntarily participated in the program as local committee members. The program comprised case management according to individual risk level and group-based activities; the protocol is detailed in Fig. 1.

#### Individual case management

Case management according to individual risk level (at risk, risk, or high-risk) was conducted by both CMHS staff and local committee members. The at-risk group was defined as having one or more sociodemographic risk factors (living alone, chronic medical illness, and heavy drinking), but with an SGDS-K score below 6. The risk group included those with a score of 6 or 7 on the SGDS-K and no history of suicidality (suicidal ideation, plans, or attempts), while individuals in the high-risk group had SGDS-K scores of 8 or more and/or a history of suicidality. Risk stratification was informed by the CMHS database, including the SGDS-K and suicidal risks, which had been screened in all the elderly in Gyeonggi Province (a larger administrative district including Yeoncheon) every year as a regional suicide prevention project.

A mental health nurse (CMHS staff) provided information to these volunteers about their role to monitor for the worsening of depressive symptoms in at-risk older people and then trained them in terms of building rapport and communicating with older adults and assessing their mood status and depressive symptoms. Local committee-guided care was delivered by matching a committee member with 4~5 older residents in all risk groups who lived in the close distance with the committee member. Committee members visited the older individuals as often as possible (at least once a week). As committee members and participants were old neighbors, natural conversation on the topics of daily life or asking after each other were encouraged instead of any structured interview. They used “mood stickers” depicting facial expressions corresponding to “good”, “so-so”, or “bad”. Older adults who indicated a worsening mood or suicidal ideation were quickly referred to the CMHS team, who also engaged in case management of the older adults. According to their protocol, the higher the risk of suicide, the more intensive was the management, which could include frequent face-to-face visits. High-risk individuals were also referred to the psychiatric service, if deemed necessary (Fig. 1).

The local committee members and CMHS team had biweekly meetings, at which the local committee-guided care supervised by the CMHS team and difficult cases were discussed. Individuals in charge of other community services, such as community nurses, social service workers, and policemen, were also invited to the meetings, with the aim of providing additional community services in certain cases (e.g., a disabled elderly man with poor nutrition was referred to a community meal program, and a woman living alone was registered on the 911 service and an emergency alarm system was installed in her home).

#### Group-based program

The group-based program involved eight weekly sessions, which were mainly aimed at enhancing interpersonal networks and community cohesion; the sessions were open to all community-dwelling older adults, regardless of risk status. The CMHS staff supervised the 2-h-long sessions, which included 15 to 20 attendees. The first half of the group program (sessions 1–4) was used to match participants each other, allow them to get to know each other, and extend their social networks; during the second half of the program (sessions 5–8), the participants acquired skills to be aware of at-risk neighbors and to encourage help-seeking (Fig. 1).

### Active control

In the control group, the CMHS provided standard care for high-risk older adults who had suicidal ideation or severe depressive symptoms (SGDS-K ≥ 10) based on the CMHS database; high-risk elderly participants were referred to the case management team and the CMHS staff met with them once a month on average. At the end of the study, we introduced the control group to the intervention program.

### Measures

Blinded researchers evaluated the patients’ demographics, clinical data, depressive symptoms, and suicidality, at baseline and at the end of the program; some of these assessments were conducted by the CMHS team.

#### Primary outcomes

We assessed changes in SGDS-K scores as the primary outcome. Scores on the SGDS-K are highly correlate with those on the original GDS, such that it has high internal consistency, and high content and differential validity [17]. SGDS-K scores corresponding to severe depression (≥ 8) were also noted [17]. Major depressive disorder, based on the DSM-IV criteria [18], was also assessed in all participants during a structured clinical interview using the Korean version of the Composite International Diagnostic Interview (K-CIDI) [19]. Suicidality (suicidal ideation, plans, or attempts) was examined using the Suicide Prevention Multisite Intervention Study on Suicidal Behaviors (SUPRE-MISS) [20].

#### Secondary outcomes

Secondary outcomes included social networks, functional status, and global cognition. We used a validated Korean version of the Lubben Social Network Scale (LSNS-K), which was developed specifically for older adults, to assess the quantity and frequency of participants’ social interactions with their relatives and friends [21, 22]. Daily functional status was evaluated using the Seoul-Instrumental Activities of Daily Living (S-IADL) instrument [23]. and global cognitive function was assessed using the Korean version of the Mini-Mental State Examination (MMSE-KC), which is part of the neuropsychological battery of the Consortium to Establish a Registry for Alzheimer’s Disease (CERAD-K) [24].

### Data analysis

Baseline characteristics were compared between groups using Student’s *t*-test for continuous variables and the chi-square test for categorical variables. The present study employed an intent-to-treat approach that included all participants who completed the baseline assessments. Linear mixed-effects models including the factors of intervention group (reference: control), time (reference: baseline), and their interaction, were used to examine group differences in changes in primary and secondary outcomes from baseline to follow-up. The models were adjusted for age, sex, years of education, and type of medical insurance (Medicare or Medicaid). Logistic regression models were used to analyze severe depressive symptoms (SGDS-K ≥ 8; indicative of major depression) [17], DSM-defined major or minor depression, and suicidality outcomes at the follow-up assessment by group. All models were adjusted for age, sex, years of education, type of medical insurance, and baseline outcomes. We repeated these analyses after dividing the participants into low (SGDS-K <  6) and high (SGDS-K ≥ 6) depressive symptom score groups at baseline.

All analyses were conducted using SPSS software (ver. 25.0; SPSS Inc., Chicago, IL, USA). All statistical tests were two-tailed, and *p* < 0.05 was deemed to indicate statistical significance.

## Results

**Fig. 2:**
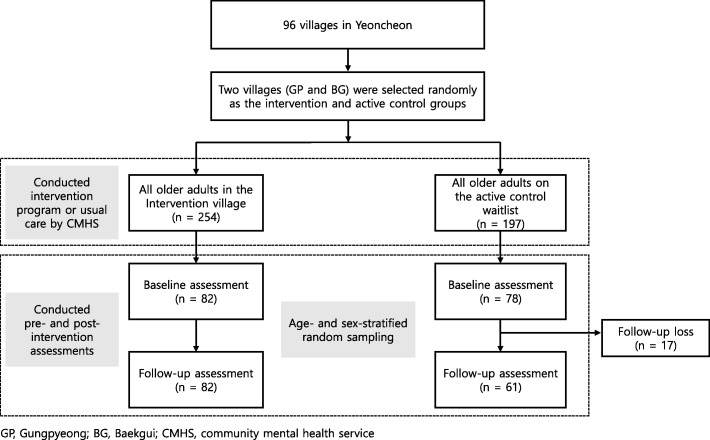
Flowchart of study enrollment, group allocation, and assessment. GP, Gungpyeong; BG, Baekgui; CMHS, community mental health service

Of all older adults living in the two villages (*n* = 451), 160 (35.5%; intervention, *n* = 82; control, *n* = 78) were included in the assessments. Figure 2 shows a flowchart of participant enrollment and assessment; enrollment continued until the target sample size was achieved. During the study period, 17 individuals in the control group were lost to follow-up due to poor health, lack of time, or relocation to another area. No adverse events were reported during the study period.

Table 1 shows the baseline sociodemographic and clinical characteristics of the participants by group. Overall, the participants had a mean (SD) age of 74.2 (5.7) years and 5.1 (4.1) years of education; 94 (55.8%) were female. Older adults living in the intervention village were less educated and had poorer socioeconomic status, reflected by greater their higher rate of Medicaid coverage; they also had a higher rate of lifetime history of suicidality at baseline, higher baseline depressive symptom scores, and lower baseline social network scores compared with those in the control group.

**Table 1:** Baseline sociodemographic and clinical characteristics

	Total (***n*** = 160)	Intervention (***n*** = 82)	Control (***n*** = 78)	p^*^
**Sociodemographic characteristics**				
Age, years ± SD	74.2 ± 5.7	74.9 ± 5.4	73.5 ± 5.9	0.120
Female, n (%)	94 (58.8)	50 (61.0)	44 (56.4)	0.631
Education, years ± SD	5.1 ± 4.1	4.3 ± 3.9	5.9 ± 4.2	0.013
Widowed/Divorced/Separated/Unmarried, n (%)	38 (24.2)	16 (20.3)	22 (28.2)	0.268
Living alone, n (%)	32 (20.4)	13 (16.0)	19 (25.0)	0.173
Medicaid, n (%)	29 (18.1)	20 (24.4)	9 (11.5)	0.041
**Clinical characteristics**				
**Lifetime**				
History of depressive disorder, ^a^ n (%)	38 (24.2)	24 (30.4)	14 (17.9)	0.093
History of suicidal ideation/plans/attempts, n (%)	32 (21.8)	21 (30.0)	11 (14.3)	0.028
**Baseline**				
Chronic medical illness, ^b^ n (%)	112 (80.6)	66 (83.5)	46 (76.7)	0.388
Depressive disorder, ^a^ n (%)	15 (9.6)	10 (12.7)	5 (6.4)	0.277
Suicidal ideation/plans/attempts, n (%)	8 (5.5)	7 (10.4)	1 (1.3)	0.025
SGDS-K, ^c^ score ± SD	3.0 ± 3.4	3.6 ± 3.7	2.4 ± 3.0	0.027
LSNS-K, ^d^ score ± SD	25.8 ± 9.2	23.1 ± 8.5	28.5 ± 9.1	< 0.001
S-IADL, ^e^ score ± SD	2.2 ± 3.7	2.4 ± 3.7	2.0 ± 3.7	0.482
MMSE-KC, ^f^ score ± SD	25.6 ± 4.6	24.9 ± 5.3	26.2 ± 3.7	0.072

^*^ Student’s *t*-test for continuous variables and the chi-square test for categorical variables

^a^ Major or minor depressive episode as defined by the DSM-IV criteria

^b^ One or more chronic medical illnesses including hypertension, diabetes, dyslipidemia, cerebrovascular disease, and heart disease

^c^ Korean version of the Geriatric Depression Scale – Short Form; a higher score corresponds to more depressive symptoms (range: 0–15)

^d^ Korean version of the Lubben Social Network Scale; a higher score corresponds to a stronger social network (range: 0–50)

^e^ Seoul Instrumental Activities of Daily Living; a higher score corresponds to worse daily function (range: 0–45)

^f^ Mini-Mental State Examination; part of the neuropsychological battery of the Consortium to Establish a Registry for Alzheimer’s Disease; a higher score corresponds to better global cognitive function (range: 0–30)

The linear mixed-effects models did not show a significant group × time interaction for the primary outcome measure (Table 2). By contrast, among individuals with high depressive symptom scores at baseline (SGDS-K ≥ 6), a greater decrease was observed in the intervention group than in the controls, although the difference did not reach significance (mean difference [SD], − 2.41 [3.83] and − 0.77 [2.59], respectively; β [SE] = 3.05 [2.86], t (28) 1.066, *p* = 0.296).

**Table 2:** Linear mixed-effects models ^a^ of the effects of group and time, and their interaction, on the outcome variables

	Primary outcome			Secondary outcomes		
	SGDS-K ^b^			LSNS-K ^d^	S-IADL ^e^	MMSE-KC ^f^
	Total	Baseline fewer depressive symptoms ^c^	Baseline more depressive symptoms ^c^			
Main effect of group	5.00* (1, 152)	9.55 (1, 119)	2.04 (1, 28)	8.18* (1, 153)	0.04 (1, 153)	1.76 (1, 153)
Main effect of time	0.08 (1, 151)	7.00 (1, 119)	4.58* (1, 28)	8.38* (1, 153)	5.48* (1, 153)	1.91 (1, 153)
Group × time interaction effect	0.49 (1, 151)	5.85* (1, 119)	1.14 (1, 28)	5.29* (1, 153)	0.55 (1, 153)	0.25 (1, 153)

*p < 0.05

^a^ The linear mixed models included the factors of group and time, and the group × time interaction, with adjustment for age, sex, years of education, and type of medical insurance. Data are F statistics (numerator and denominator degrees of freedom)

^b^ Korean version of the Geriatric Depression Scale – Short Form; a higher score corresponds to more depressive symptoms (range: 0–15)

^c^ Participants were stratified by baseline SGDS-K scores (< 6 vs. ≥ 6)

^d^ Korean version of the Lubben Social Network Scale; a higher score corresponds to a stronger social network (range: 0–50)

^e^ Seoul Instrumental Activities of Daily Living; a higher score corresponds to worse daily function (range: 0–45)

^f^ Mini-Mental State Examination; part of the neuropsychological battery of the Consortium to Establish a Registry for Alzheimer’s Disease; a higher score corresponds to better global cognitive function (range: 0–30)

Rates of severe depression (SGDS-K ≥ 8), incident major or minor depressive episode, and incident suicidality did not differ between the intervention and control groups at post-intervention period. However, in the logistic regression analysis dividing subjects into low and high baseline depressive symptom groups, trends toward a decreased risk of severe depression (SGDS K ≥ 8) and incident major or minor depressive episodes were observed in intervention group subjects with more depressive symptoms at baseline (Table 3).

**Table 3:** Logistic regression analyses of the associations of severe depressive symptoms, depressive disorder, and suicidality outcomes with the intervention (vs. active controls) at the follow-up assessment

	Total sample			Baseline fewer depressive symptoms ^a^			Baseline more depressive symptoms ^a^		
	Intervention	Control	Adjusted OR^c^ (95% CI)	Intervention	Control	Adjusted OR^c^ (95% CI)	Intervention	Control	Adjusted OR^c^ (95% CI)
	case/n^b^ (%)			case/n^b^ (%)			case/n^b^ (%)		
**Severe depressive symptoms**									
SGDS ≥8	16/81 (19.8)	5/60 (8.3)	2.90 (0.68–12.46)	6/58 (10.3)	0/50 (0.0)	_	10/19 (52.6)	5/10 (50.0)	0.02 (0.00–17.47)
**Depressive disorder**									
Major or minor depressive episode	5/77 (6.5)	2/60 (3.3)	1.38 (0.21–8.83)	2/55 (3.6)	0/51 (0.0)	_	3/18 (16.7)	2/9 (22.2)	0.48 (0.04–6.02)
**Suicidality**									
Suicidal ideation/plans/attempts	5/57 (8.8)	2/60 (3.3)	2.24 (0.31–16.04)	3/41 (7.3)	1/50 (2.0)	4.98 (0.37–67.69)	2/12 (16.7)	1/10 (10.0)	_

*OR* Odds ratio, *CI* Confidence interval, *SGD* Geriatric Depression Scale – Short Form

^a^ Participants were stratified by baseline SGDS scores (< 6 vs. ≥ 6)

^b^ Numbers of participants followed-up for each outcome variable

^c^ Odds ratios with active controls as the reference group: adjusted for age, sex, years of education, type of medical insurance, and baseline outcome variables

Regarding secondary outcomes, a significant group × time interaction for scale of social network as seen in the intervention group (Table 2). *Post-hoc* analyses showed a greater increase in the LSNS-K in the intervention group between baseline and follow-up (mean difference [SD], 3.67 [7.32]) than in the control group (0.53 [10.61]; β [SE] = 3.08 [1.34], t (153) = 2.301, *p* = 0.023). No other significant differences were observed between groups.

## Discussion

The village-based intervention for late-life depression conducted in this study did not reduce depressive symptoms in older adults overall; however, the program tended to lower the risk of progression to severe depression among at-risk older adults, and increased the social network more than five-fold compared with standard CMHS care.

To our knowledge, this is the first multilevel depression intervention for community-dwelling older adults using representative samples. Most previous community-based depression or suicide interventions measured the effectiveness of their protocols according to health-related indices or suicide rates at the general population level [25–29]. They were generally effective in improving mental health indices (e.g., rates of mental health service use) or lowering overall suicide rates; however, the measures deemed most important, and how the protocols affected the at-risk population specifically, were unclear. We assessed depression and suicidality in participants directly, according to demographic and clinical factors both before and after the intervention. The results suggest that our community-based protocol was effective for increasing social bonding in older adults although depressive symptoms were not reduced by the intervention: it only showed a tendency of preventing the progression to severe depression in the at-risk population.

Our 12-week intervention did not reduce depressive symptoms in the older population overall, which was probably due to the inclusion of large numbers of healthy elderly individuals. Based on growing evidence that multilevel approaches are more effective for managing depression and reducing suicide rates [11, 30–32], we used such a design in our community-based intervention to prevent late-life depression in a healthy population and at-risk individuals. However, the primary outcome (changes in depressive symptoms) might not have been sufficient to demonstrate the benefits of our short-term program, because there was little room to for improvement of SGDS-K scores given the low average score at baseline. On the other hand, although our program was open to all elderly residents of the intervention village, the pre- and post-intervention assessments were performed only in representative samples, after excluding individuals who had difficulty participating in the interview. Therefore, it is possible that the effectiveness of our intervention was underestimated due to exclusion of the data for unhealthy older adults who underwent the intervention but were not analysed. Additionally, although the older adults in the study generally exhibited good cognitive health, even mild impairments in cognitive function could have influenced the reports of depression. This was especially true for K-CIDI-defined depressive disorders based on DSM-IV criteria, because this tool uses somewhat complex questions. Although additional adjustments, including for baseline MMSE-KC scores, did not change the primary results of the logistic regression models, the inclusion of clinical assessments of depressive disorders by psychiatrists should be considered in future studies.

Meanwhile, there was an even increased tendency for severe types of depression to be reported in the intervention group. It is possible that this type of disclosure was more common because baseline SGDS-K scores were significantly higher in the intervention group than the active control group, even though the scores were within the normal range for both groups. Additionally, older adults in the intervention group might have been more likely to report their mental health problems after interacting with the local committee members and/or the CMHS staff. In general, older Asians are unwilling to disclose their mental health problems to other people because they tend to perceive emotional problems as a sign of weakness, a lack of discipline, and/or a lack of willpower [33]. With considerations for this cultural context, we cautiously suggest that the participants in the intervention group might have been less reluctant than the active controls to report their severe depression to the researchers after adapting to expressing their emotional status during the intervention program.

In the present study, there was a tendency for the intervention to prevent the progression of severe depression in at-risk elderly individuals (i.e., those with moderately high depressive symptoms at baseline). These results suggest that a more targeted intervention for at-risk older adults would be beneficial, even though the results did not reach the level of significance. This finding is consistent with those of previous community- and clinic-based trials targeting individuals at high risk of depression. However, GPs were mainly responsible for screening patients for depression and referring them for psychiatric treatment in most such studies [9, 10, 34]. There are few medical services in many rural areas of Korea, including Yeoncheon, in which only 29 physicians and one psychiatrist cover a population of 44,187 [16]. Moreover, a primary care system involving GPs is not the standard in many non-Western countries, including Korea. Therefore, our protocol has a possibility to be particularly useful for managing high-risk older adults living in small rural communities, although further studies including more numbers of villages are needed.

Our community-based intervention for geriatric depression strengthened the interpersonal networks of elderly individuals, as measured by the LSNS-K; this scale evaluates not only the size of an individual’s social network, but also the frequency and feasibility of networking. A lack of social relationships is associated with various health problems [35, 36], including depression [37–39] and even mortality [40, 41]. Depressed older adults tend to have more fragile social networks, which was reported to be related to a greater propensity toward suicidal thoughts [42]. A previous community intervention that used a group-based approach to increase the social networks of older adults showed promising results [43], consistent with our study.

Social support may protect older adults against worsening depression, by mitigating psychosocial stressors [37]. A large community-based observational study showed that infrequent social interactions was significantly associated with depression in older adults [39]. People typically have less contact with friends and colleagues as they age, so it is important to re-establish interpersonal relationships, especially with close friends or neighbors [43]. Our program aimed to strengthen the autonomy of village-dwellers; healthy local committee members were responsible for monitoring the at-risk older adults, and group activities were used to encourage reciprocal care. Encouraging older adults to assume active roles in the community might increase their social networks. Our promising intervention should be assessed in the context of long-term prevention of late-life depression in future studies.

This study had several limitations. First, although we randomly allocated two villages as the intervention and active control, two group sizes may restrict the conclusions. Second, while all elderly population living in two villages were targeted by intervention or usual care, respectively, only one third of them participated in the baseline and follow-up interviews even though they were randomly selected from each group as representative populations. Third, the blinding of both researchers and participants was only partial. Lastly, the intervention was conducted over a short period compared with other depression prevention programs.

## Conclusion

Our village-based intervention for late-life depression, led by healthy community residents and CMHS staff, was effective in increasing the social networks of older adults and might prevent severe depression among at-risk elderly individuals. We believe that this protocol is potentially useful for preventing depression and suicide in older adults living in small rural communities, which usually have insufficient medical services. Future studies that include more villages, employ a long-term follow-up period, and apply more targeted strategies for at-risk older adults will be needed to validate the findings of the present feasibility study.

## Data Availability

The datasets used and/or analysed during the current study are available from the corresponding author on reasonable request.

## Abbreviations

CERAD-K: The neuropsychological battery of the Consortium to Establish a Registry for Alzheimer’s Disease
CMHS: Community mental health service
GP: General physician
K-CIDI: Korean version of the Composite International Diagnostic Interview
LSNS-K: Korean version of the Lubben Social Network Scale
MMSE-KC: Korean version of the Mini-Mental State Examination
SGDS-K: Korean version of the Geriatric Depression Scale – Short Form
S-IADL: Seoul-Instrumental Activities of Daily Living
SUPRE-MISS: Suicide Prevention Multisite Intervention Study on Suicidal Behaviors
